# Spectral stability of periodic waves in the generalized reduced Ostrovsky equation

**DOI:** 10.1007/s11005-017-0941-3

**Published:** 2017-02-02

**Authors:** Anna Geyer, Dmitry E. Pelinovsky

**Affiliations:** 1 Delft Institute of Applied Mathematics, Faculty Electrical Engineering, Mathematics and Computer Science, Delft University of Technology, Mekelweg 4, 2628 CD Delft, The Netherlands; 2 Department of Mathematics, McMaster University, Hamilton, ON L8S 4K1 Canada; 3 Department of Applied Mathematics, Nizhny Novgorod State Technical University, Nizhny Novgorod, 603950 Russia

**Keywords:** Reduced Ostrovsky equations, Stability of periodic waves, Energy-to-period map, Negative index theory, 35B35, 35G30

## Abstract

We consider stability of periodic travelling waves in the generalized reduced Ostrovsky equation with respect to co-periodic perturbations. Compared to the recent literature, we give a simple argument that proves spectral stability of all smooth periodic travelling waves independent of the nonlinearity power. The argument is based on the energy convexity and does not use coordinate transformations of the reduced Ostrovsky equations to the semi-linear equations of the Klein–Gordon type.

## Introduction

We address the generalized reduced Ostrovsky equation written in the form

1 $$\begin{aligned} (u_t + u^p u_x)_x = u, \end{aligned}$$

where $p \in {\mathbb {N}}$ is the nonlinearity power and *u* is a real-valued function of (*x*, *t*). This equation was derived in the context of long surface and internal gravity waves in a rotating fluid for $p = 1$ [22] and $p = 2$ [7]. These two cases are the only cases, for which the reduced Ostrovsky equation is transformed to integrable semi-linear equations of the Klein–Gordon type by means of a change of coordinates [6, 14].

We consider existence and stability of travelling periodic waves in the generalized reduced Ostrovsky equation (1) for any $p \in {\mathbb {N}}$. The travelling 2*T*-periodic waves are given by $u(x,t) = U(x-ct)$, where $c > 0$ is the wave speed, *U* is the wave profile satisfying the boundary value problem

2 $$\begin{aligned} \frac{\mathrm{d}}{\mathrm{d}z} \left[ (c - U^p) \frac{\mathrm{d}U}{\mathrm{d}z} \right] + U(z) = 0, \quad U(-T) = U(T), \quad U'(-T) = U'(T), \end{aligned}$$

and $z = x-ct$ is the travelling wave coordinate. We are looking for smooth periodic waves $U \in H^{\infty }_{\mathrm{per}}(-T,T)$ satisfying (2). It is straightforward to check that periodic solutions of the second-order equation (2) correspond to level curves of the first-order invariant,

3 $$\begin{aligned} E = \frac{1}{2} (c - U^p)^2 \left( \frac{\mathrm{d}U}{\mathrm{d}z} \right) ^2 + \frac{c}{2} U^2 - \frac{1}{p+2} U^{p+2} = \mathrm{const}. \end{aligned}$$

We add a *co-periodic* perturbation to the travelling wave, that is, a perturbation with the same period 2*T*. Separating the variables, the spectral stability problem for the perturbation *v* to *U* is given by $\lambda v = \partial _z L v$, where

4 $$\begin{aligned} L = P_0 \left( \partial _z^{-2} + c - U(z)^p \right) P_0{:} \; {\dot{L}}^2_{\mathrm{per}}(-T,T) \rightarrow {\dot{L}}^2_{\mathrm{per}}(-T,T), \end{aligned}$$

where ${\dot{L}}^2_{\mathrm{per}}(-T,T)$ denotes the space of 2*T*-periodic, square-integrable functions with zero mean and $P_0{:}\,L^2_{\mathrm{per}}(-T,T) \rightarrow {\dot{L}}^2_{\mathrm{per}}(-T,T)$ is the projection operator that removes the mean value of 2*T*-periodic functions.

### Definition 1

We say that the travelling wave is spectrally stable with respect to co-periodic perturbations if the spectral problem $\lambda v = \partial _z L v$ with $v \in {\dot{H}}^1_{\mathrm{per}}(-T,T)$ has no eigenvalues $\lambda \notin i {\mathbb {R}}$.

Local solutions of the Cauchy problem associated with the generalized reduced Ostrovsky equation (1) exist in the space ${\dot{H}}^s_{\mathrm{per}}(-T,T)$ for $s > \frac{3}{2}$ [26]. For sufficiently large initial data, the local solutions break in finite time, similar to the inviscid Burgers equation [18, 19]. However, if the initial data $u_0$ is small in a suitable norm, then local solutions are continued for all times in the same space, at least in the integrable cases $p = 1$ [8] and $p = 2$ [25].

Travelling periodic waves to the generalized reduced Ostrovsky equation (1) were recently considered in the cases $p = 1$ and $p = 2$. In these cases, travelling waves can be found in the explicit form given by the Jacobi elliptic functions after a change of coordinates [6, 14]. Exploring this idea further, it was shown in [10, 11, 27] that the spectral stability of travelling periodic waves can be studied with the help of the eigenvalue problem $M \psi = \lambda \partial _z \psi$, where *M* is a second-order Schrödinger operator. Independently, by using higher-order conserved quantities which exist in the integrable cases $p = 1$ and $p = 2$, it was shown in [15] that the travelling periodic waves are unconstrained minimizers of energy functions in suitable function spaces with respect to *subharmonic* perturbations, that is, perturbations with a multiple period to the periodic waves. This result yields not only spectral but also nonlinear stability of the travelling wave. The nonlinear stability of periodic waves was established analytically for small-amplitude waves and shown numerically for waves of arbitrary amplitude [15].

In this paper, we give a simple argument that proves spectral stability of all smooth periodic travelling waves to the generalized reduced Ostrovsky equation (1) independently of the nonlinearity power *p* and the wave amplitude. The spectral stability of periodic waves is defined here with respect to co-periodic perturbations in the sense of Definition 1. The argument is based on convexity of the energy function

5 $$\begin{aligned} H(u) = -\frac{1}{2} \Vert \partial _x^{-1} u \Vert _{L^2_{\mathrm{per}}}^2 - \frac{1}{(p+1)(p+2)} \int _{-T}^T u^{p+2} \mathrm{d}x, \end{aligned}$$

at the travelling wave profile *U* in the energy space with fixed momentum,

6 $$\begin{aligned} X_q = \left\{ u \in {\dot{L}}^2_{\mathrm{per}}(-T,T) \cap L^{p+2}_{\mathrm{per}}(-T,T){:} \quad \Vert u \Vert ^2_{L^2_{\mathrm{per}}} = 2 q > 0 \right\} . \end{aligned}$$

Note that the self-adjoint operator *L* given by (4) is the Hessian operator of the extended energy function $F(u)=H(u) + c Q(u)$, where

7 $$\begin{aligned} Q(u) = \frac{1}{2} \Vert u \Vert ^2_{L^2_{\mathrm{per}}} \end{aligned}$$

is the momentum function. The energy *H*(*u*) and momentum *Q*(*u*), and therefore the extended energy *F*(*u*), are constants of motion, as can be seen readily by writing the evolution equation (1) in Hamiltonian form as $u_t = \partial _x \mathrm{grad} H(u)$. Notice that the travelling wave profile *U* is a critical point of the extended energy function *F*(*u*) in the sense that the Euler–Lagrange equations for *F*(*u*) are identical to the boundary value problem (2) after the second-order equation is integrated twice with zero mean.

The outline of the paper is as follows. Adopting the approach from [3–5], we prove in Sect. 2 that the energy-to-period map $E \mapsto 2T$ is strictly monotonically decreasing for the family of smooth periodic solutions satisfying (2) and (3). This result holds for every fixed $c > 0$. Thanks to monotonicity of the energy-to-period map $E \mapsto 2T$, the inverse mapping defines the first-order invariant *E* in terms of the half period *T* and the speed *c*. We denote this inverse mapping by *E*(*T*, *c*).

In Sect. 3, we consider continuations of the family of smooth periodic solutions with respect to parameter *c* for every fixed $T > 0$ and prove that *E*(*T*, *c*) is an increasing function of *c* within a nonempty interval $(c_0(T),c_1(T))$, where $0< c_0(T)< c_1(T) < \infty$. We also prove that the momentum *Q*(*u*) evaluated at $u = U$ is an increasing function of *c* for every fixed $T > 0$.

In Sect. 4, we use the monotonicity of the mapping $E \mapsto 2T$ for every fixed $c > 0$ and prove that the self-adjoint operator *L* given by (4) has a simple negative eigenvalue, a one-dimensional kernel, and the rest of its spectrum is bounded from below by a positive number.

Finally, in Sect. 5, we prove that the operator *L* constrained on the space

8 $$\begin{aligned} L^2_c = \left\{ u \in {\dot{L}}^2_{\mathrm{per}}(-T,T){:} \quad \langle U, u \rangle _{L^2_{\mathrm{per}}} = 0 \right\} \end{aligned}$$

is strictly positive except for the one-dimensional kernel induced by the translational symmetry. This gives convexity of *H*(*u*) at $u = U$ in space of fixed *Q*(*u*) given by (6). By using the standard Hamilton–Krein theorem in [12] (see also the reviews in [17, 24]), this rules out existence of eigenvalues $\lambda \notin i {\mathbb {R}}$ of the spectral problem $\lambda v = \partial _z L v$ with $v \in {\dot{H}}^1_{\mathrm{per}}(-T,T)$.

All together, the existence and spectral stability of smooth periodic travelling waves of the generalized reduced Ostrovsky equation (1) is summarized in the following theorem.

### Theorem 1

For every $c > 0$ and $p \in {\mathbb {N}}$,

- there exists a smooth family of periodic solutions $U \in {\dot{L}}^2_{\mathrm{per}}(-T,T) \cap H^{\infty }_{\mathrm{per}}(-T,T)$ of Eq. (2), parameterized by the energy *E* given in (3) for $E \in (0,E_c)$, with
  $$\begin{aligned} E_c = \frac{p}{2(p+2)} c^{\frac{p+2}{p}}, \end{aligned}$$
  such that the energy-to-period map $E \mapsto 2T$ is smooth and strictly monotonically decreasing. Moreover, there exists $T_1 \in (0,\pi )$ such that
  $$\begin{aligned} T \rightarrow \pi c^{\frac{1}{2}} \quad \text{ as } \quad E \rightarrow 0 \quad \text{ and } \quad T \rightarrow T_1 c^{\frac{1}{2}} \quad \text{ as } \quad E \rightarrow E_c; \end{aligned}$$
- for each point *U* of the family of periodic solutions, the operator *L* given by (4) has a simple negative eigenvalue, a simple zero eigenvalue associated with $\mathrm{Ker}(L) = \mathrm{span}\{\partial _z U \}$, and the rest of the spectrum is positive and bounded away from zero;
- the spectral problem $\lambda v = \partial _z Lv$ with $v \in {\dot{H}}^1_{\mathrm{per}}(-T,T)$ admits no eigenvalues $\lambda \notin i {\mathbb {R}}$.

Consequently, periodic waves of the generalized reduced Ostrovsky equation (1) are spectrally stable with respect to co-periodic perturbations in the sense of Definition 1.

We now compare our result to the existing literature on spectral and orbital stability of periodic waves with respect to co-periodic perturbations. First, in comparison with the analysis in [11], the result of Theorem 1 is more general since $p \in {\mathbb {N}}$ is not restricted to the integrable cases $p = 1$ and $p = 2$. On a technical level, the method of proof of Theorem 1 is simple and robust, so that many unnecessary explicit computations from [11] are avoided. Indeed, in the transformation of the spectral problem $\lambda v = \partial _z L v$ to the spectral problem $M \psi = \lambda \partial _z \psi$, where *M* is a second-order Schrödinger operator from $H^2_{\mathrm{per}}(-T,T) \rightarrow L^2_{\mathrm{per}}(-T,T)$, the zero-mean constraint is lost.^1^ Consequently, the operator *M* was found in [11] to admit two negative eigenvalues in $L^2_{\mathrm{per}}(-T,T)$, which are computed explicitly by using eigenvalues of the Schrödinger operator with elliptic potentials. By adding three constraints for the spectral problem $M \psi = \lambda \partial _z \psi$, the authors of [11] showed that the operator *M* becomes positive on the constrained space, again by means of symbolic computations involving explicit Jacobi elliptic functions. All these technical details become redundant in our simple approach.

Second, we mention another type of improvement of our method compared to the analysis of spectral stability of periodic waves in other nonlinear evolution equations [20, 21]. By establishing first the monotonicity of the energy-to-period map $E \mapsto 2T$ for a smooth family of periodic waves, we give a very precise count on the number of negative eigenvalues of the operator *L* in $L^2_{\mathrm{per}}(-T,T)$ without doing numerical approximations on solutions of the homogeneous equation $L v = 0$. Indeed, the smooth family of periodic waves has a limit to zero solution, for which eigenvalues of *L* in $L^2_{\mathrm{per}}(-T,T)$ are found from Fourier series. The zero eigenvalue of *L* is double in this limit and it splits once the amplitude of the periodic wave becomes nonzero. Owing to the monotonicity of the map $E \mapsto 2T$ and continuation arguments, the negative index of the operator *L* remains invariant along the entire family of the smooth periodic waves. Therefore, the negative index of the operator *L* is found for the entire family of periodic waves by a simple argument, again avoiding cumbersome analytical or approximate numerical computations.

Finally, we also mention that the spectral problem $\lambda v = \partial _z L v$ is typically difficult when it is posed in the space $L^2_{\mathrm{per}}(-T,T)$ because the mean-zero constraint is needed on *v* in addition to the orthogonality condition $\langle U, v \rangle _{L^2_{\mathrm{per}}} = 0$. The two constraints are taken into account by studying the two-parameter family of smooth periodic waves and working with a 2-by-2 matrix of projections [1, 16]. This complication is avoided for the reduced Ostrovsky equation (1) because the spectral problem $\lambda v = \partial _z L v$ is posed in space ${\dot{L}}^2_{\mathrm{per}}(-T,T)$ and the only orthogonality condition $\langle U, v \rangle _{L^2_{\mathrm{per}}} = 0$ is studied with the help of identities satisfies by the periodic wave *U*.

As a limitation of the results of Theorem 1, we mention that the nonlinear orbital stability of travelling periodic waves cannot be established for the reduced Ostrovsky equations (1) by using the energy function (5) in space (6). This is because the local solution is defined in ${\dot{H}}^s_{\mathrm{per}}(-T,T)$ for $s > \frac{3}{2}$ [26], whereas the energy function is defined in ${\dot{L}}^2_{\mathrm{per}}(-T,T) \cap L^{p+2}_{\mathrm{per}}(-T,T)$. As a result, coercivity of *H*(*u*) in the space of fixed momentum (6) only controls the $L^2$ norm of time-dependent perturbations. Local well-posedness in such spaces of low regularity is questionable and so is the proof of orbital stability of the travelling periodic waves in the time evolution of the reduced Ostrovsky equations (1).

## Monotonicity of the energy-to-period map

**Fig. 1:**
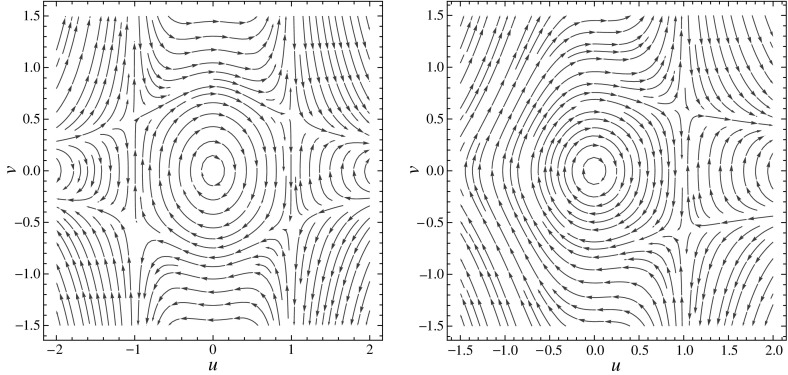
Phase portraits of system (9) for $p = 2$ (*left*) and $p = 1$ (*right*)

Travelling wave solutions of the reduced Ostrovsky equation (1) are solutions of the second-order differential equation (2) with fixed $c > 0$ and $p \in {\mathbb {N}}$. The following lemma establishes a correspondence between the smooth periodic solutions of the second-order equation (2) and the periodic orbits around the centre of an associated planar system; see Fig. 1. For lighter notations, we replace *U*(*z*) by *u*(*z*) and denote the derivatives in *z* by primes.

### Lemma 1

For every $c>0$ and $p\in {\mathbb {N}}$ the following holds:

(i) A function *u* is a smooth periodic solution of Eq. (2) if and only if $(u,v)=(u,u')$ is a periodic orbit of the planar differential system
  9 $$\begin{aligned} \left\{ \begin{array}{l} u' =v,\\ v' =\dfrac{-u + pu^{p-1}v^2}{c-u^p}. \end{array}\right. \end{aligned}$$
(ii) The system (9) has a first integral given by (3), which we write as
  10 $$\begin{aligned} E(u,v)=A(u)+B(u) v^2, \end{aligned}$$
  with $$A(u) = \frac{c}{2}u^2- \frac{1}{p+2} u^{p+2}$$ and $$B(u) = \frac{1}{2}(c-u^p)^2$$.
(iii) Every periodic orbit of system (9) belongs to the period annulus^2^ of the centre at the origin of the (*u*, *v*) plane and lies inside some energy level curve of *E*, with $E \in (0,E_c)$ where
  11 $$\begin{aligned} E_c := A(c^{1/p}) = \frac{p}{2(p+2)} c^{\frac{p+2}{p}}. \end{aligned}$$

### Proof

The assertion in (*ii*) is proved with a straightforward calculation. To prove (*iii*), we notice that system (9) has no limit cycles in view of the existence of a first integral, and hence the periodic orbits form period annuli. A periodic orbit must surround at least one critical point. The unique critical point of system (9) is a centre at the origin on the (*u*, *v*) plane, corresponding to the energy level $E =0$. In view of the presence of the singular line

$$\begin{aligned} \left\{ u = c^{1/p}, \quad v \in {\mathbb {R}}\right\} \subset {\mathbb {R}}^2 \end{aligned}$$

we may conclude, applying the Poincaré–Bendixon Theorem, that the set of periodic orbits forms a punctured neighbourhood of the centre and that no other period annulus is possible.

It remains to show (*i*). It is clear that $z \mapsto (u,v)=(u,u')$ is a smooth solution of the differential system (9) if and only if *u* is a smooth solution of the second-order equation (2) satisfying $c\ne u(z)^p$ for all *z*. We claim that $c\ne u(z)^p$ for all $z\in {\mathbb {R}}$ for smooth periodic solutions *u*. Indeed, let *p* be odd for simplicity and recall that every periodic orbit in a planar system has exactly two turning points $(u,u')=(u_{\pm },0)$ per fundamental period. The turning points correspond to the maximum and minimum of the periodic solution *u* and satisfy the equation $A(u_{\pm })=E$. The graph of *A*(*u*) on ${\mathbb {R}}^+$ has a global maximum at $u=c^{1/p}$ with $E_c$ given in (11).

The equation $A(u)=E$ has exactly two positive solutions for $E\in (0,E_c)$, where $u=u_+$ corresponds to the smaller one inside the period annulus. At $E=E_c$, the equation $A(u) = E$ has only one positive solution given by $u_+=c^{1/p}$. Now assume that for a smooth periodic solution *u*, there exists $z_1$ such that $u(z_1)=c^{1/p}$. Then, Eq. (2) implies that $u'(z_1)=\pm p^{-1/2} c^{-\frac{p-2}{2p}}$; hence, the solution $(u,u')(z)$ to system (9) tends to the points $p_{\pm } = (c^{1/p},\pm p^{-1/2} c^{-\frac{p-2}{2p}})$ as $z\rightarrow z_1$. Since $E(p_{\pm })=E_c$ and by continuity of the first integral, this orbit lies inside the $E_c$-level set. For such an orbit, we have seen that its turning point is located at $u_+=c^{1/p}=u(z_1)$. However, since $u'(z_1)\ne 0$, this cannot be a turning point, which leads to a contradiction. Hence, the assertion (*i*) is proved. $\square$

### Remark 1

By Lemma 1, every smooth periodic solution *u* of the differential equation (2) corresponds to a periodic orbit $(u,v)=(u,u')$ inside the period annulus of the differential system (9). Since *E* is a first integral of (9), this orbit lies inside some energy level curve of *E*, where $E \in (0,E_c)$. We denote this orbit by $\gamma _E$. The period of this orbit is given by

12 $$\begin{aligned} 2T(E) = \int _{\gamma _E}\frac{\mathrm{d}u}{v}, \end{aligned}$$

since $\frac{\mathrm{d}u}{\mathrm{d}z} = v$ in view of (9). The energy levels of the first integral *E* parameterize the set of periodic orbits inside the period annulus, and therefore, this set forms a smooth family $\{\gamma _E\}_{E\in (0,E_c)}$. In view of Lemma 1, we can therefore assert that the set of smooth periodic solutions of (2) forms a smooth family $\{u_E\}_{E\in (0,E_c)}$, which is parameterized by *E* as well. Moreover, it ensures that the period 2*T*(*E*) of the periodic orbit $\gamma _E$ is equal to the period of the corresponding smooth periodic solution $u_E$ of the second-order equation (2).

The main result of this section is the following proposition, from which we conclude that the energy-to-period map $E \mapsto 2T(E)$ for the smooth periodic solutions of Eq. (2) is smooth and strictly monotonically decreasing. Together with Remark 1 above and Lemma 2 below, this proves statement (a) of Theorem 1.

### Proposition 1

For every $c > 0$ and $p \in {\mathbb {N}}$, the function

$$\begin{aligned} T: (0,E_c) \longrightarrow {\mathbb {R}}^+, \quad E \longmapsto T(E) = \frac{1}{2} \int _{\gamma _E}\frac{\mathrm{d}u}{v} \end{aligned}$$

is strictly monotonically decreasing and satisfies

13 $$\begin{aligned} T'(E) = -\frac{p}{4 (2+p) E} \int _{\gamma _E} \frac{u^p}{(c-u^p)}\frac{\mathrm{d}u}{v} < 0. \end{aligned}$$

### Proof

Since $A(u) + B(u) v^2=E$ is constant along an orbit $\gamma _E$, we find that

14 $$\begin{aligned} 2 E \,T(E)= \int _{\gamma _E} B(u) v \mathrm{d}u + \int _{\gamma _E} A(u) \frac{\mathrm{d}u}{v}. \end{aligned}$$

To compute the derivative of *T* with respect to *E*, we first resolve the singularity in the second integral in Eq. (14). To this end, recall that the orbit $\gamma _E$ belongs to the level curve $\{A(u) + B(u) v^2=E\}$ and therefore

15 $$\begin{aligned} \frac{\mathrm{d}v}{\mathrm{d}u} = -\frac{A'(u) + B'(u)v^2}{2B(u)v} \end{aligned}$$

along the orbit. Note that *B*(*u*) is different from zero for $$E \in (0,E_c)$$. Furthermore, $$ BA /A'$$ is bounded on $$\gamma _E$$. Using the fact that the integral of a total differential *d* over the closed orbit $$\gamma _E$$ yields zero, we find that

$$\begin{aligned} 0= & {} \int _{\gamma _E} d \left[ \left( \frac{2 BA }{A'}\right) (u) \,v \right] \\= & {} \int _{\gamma _E}\left( \frac{2 BA }{A'}\right) '(u) \,v \,\mathrm{d}u + \left( \frac{2 BA }{A'}\right) (u)\, \mathrm{d}v\\= & {} \int _{\gamma _E} \left( \frac{2 BA }{A'}\right) '(u) \,v \,\mathrm{d}u - \left( \frac{2 BA }{A'} \frac{A'}{2B}\right) (u) \frac{\mathrm{d}u}{v} - \left( \frac{2 BA }{A'} \frac{B'}{2B}\right) (u) \,v \,\mathrm{d}u\\= & {} \int _{\gamma _E} \left[ \left( \frac{2 BA }{A'}\right) '(u) -\left( \frac{ AB '}{A'}\right) (u)\right] v\, \mathrm{d}u - A(u)\frac{\mathrm{d}u}{v}, \end{aligned}$$

where we have used relation (15) in the third equality. Denoting

16 $$\begin{aligned} G = \left( \frac{2 BA }{A'} \right) ' - \frac{ AB '}{A'}, \end{aligned}$$

this ensures that

17 $$\begin{aligned} 2 ET (E) = \int _{\gamma _E} \left[ B(u) + G(u) \right] v \mathrm{d}u, \end{aligned}$$

where the integrand is no longer singular at the turning points, where the orbit $\gamma _E$ intersects with the horizontal axis $v = 0$.^3^ Taking now the derivative of Eq. (17) with respect to *E*, we obtain that

18 $$\begin{aligned} 2 T(E) + 2 E\,T'(E) = \int _{\gamma _E} \frac{B(u) + G(u)}{2B(u)v} \mathrm{d}u, \end{aligned}$$

where we have used that

$$\begin{aligned} \frac{\partial v}{\partial E} = \frac{1}{2 B(u) v} \end{aligned}$$

in view of (10).^4^ From (18), we conclude that

$$\begin{aligned} 2 T'(E)= & {} \frac{1}{E} \int _{\gamma _E} \left( \frac{B+G}{2B}\right) (u) \frac{\mathrm{d}u}{v} - \frac{1}{E} \int _{\gamma _E} \frac{\mathrm{d}u}{v} \\= & {} \frac{1}{E} \int _{\gamma _E} \frac{1}{2B} \left( \left( \frac{2 AB }{A'}\right) ' - \frac{( AB )'}{A'}\right) (u) \frac{\mathrm{d}u}{v}. \end{aligned}$$

In view of the expressions for *A* and *B* defined in Lemma 1, further calculations show that

19 $$\begin{aligned} T'(E) = -\frac{p}{4 (2+p) E} \int _{\gamma _E} \frac{u^p}{(c-u^p)}\frac{\mathrm{d}u}{v}. \end{aligned}$$

We now need to show that $T'(E) < 0$ for every $E \in (0,E_c)$. In view of the symmetry of the vector field with respect to the horizontal axis and taking into account (10), we write (19) in the form

20 $$\begin{aligned} T'(E)= & {} -\frac{p}{2 (2+p) E} \int _{u_-}^{u_+} \frac{u^p}{(c-u^p)}\sqrt{\frac{B(u)}{E-A(u)}} \mathrm{d}u \nonumber \\= & {} -\frac{p}{2 \sqrt{2} (2+p) E} \int _{u_-}^{u_+} \frac{u^p}{\sqrt{E-A(u)}} \mathrm{d}u, \end{aligned}$$

where $u_{\pm }$ denote the turning points of the orbit $\gamma _E$ with $E=A(u_{\pm })$, i.e. the intersections of the orbit $\gamma _E$ with the horizontal axis $v = 0$. Therefore, we find that $T'(E) <0$ if *p* is even. Now we show that the same property also holds when *p* is odd. Denote

21 $$\begin{aligned} I_1(E):= \int _{u_-}^0 \frac{u^p}{\sqrt{E-A(u)}} \mathrm{d}u, \quad I_2(E):= \int _0^{u_+} \frac{u^p}{\sqrt{E-A(u)}} \mathrm{d}u, \end{aligned}$$

then

22 $$\begin{aligned} T'(E)&= -\frac{p}{2 \sqrt{2}(2+p) E} \big [ I_1(E) + I_2(E)\big ]. \end{aligned}$$

We perform the change of variables $u=u_+ x$ and find that

$$\begin{aligned} I_2(E)= & {} \int _0^{u_+} \frac{u^p}{\sqrt{A(u_+)-A(u)}} \mathrm{d}u = \int _0^1 \frac{ u_+^p x^p}{\sqrt{A(u_+) - A(u_+x)}} u_+ \mathrm{d}x\\= & {} \sqrt{2}u_+^p \int _0^1 \frac{ x^p}{\sqrt{c(1-x^2) - \frac{2u_+^p }{p+2} (1-x^{p+2} )}} \mathrm{d}x. \end{aligned}$$

To rewrite the first integral, we change variables according to $u=-|u_-|x$ and obtain

$$\begin{aligned} I_1(E)= & {} \int _{-|u_-|}^0 \frac{u^p}{\sqrt{A(-|u_-|)-A(u)}} \mathrm{d}u = \int _1^0 \frac{ -|u_-|^p x^p}{\sqrt{A(-|u_-|) - A(u_-x)}} (-|u_-|) \mathrm{d}x\\= & {} -\sqrt{2}|u_-|^p \int _0^1 \frac{ x^p}{\sqrt{c(1-x^2) + \frac{2|u_-|^p }{p+2} (1-x^{p+2} )}} \mathrm{d}x. \end{aligned}$$

We claim that $|u_-| < u_+$ if *p* is odd. Indeed, we have that $A(u)<A(-u)$ on $(0,c^{1/p})$, since

$$\begin{aligned} A(u)-A(-u) = u^2 \left( \frac{c}{2} - \frac{1}{p+2} u^p \right) - u^2 \left( \frac{c}{2} + \frac{1}{p+2} u^p \right) =- \frac{2}{p+2} u^{p+2} <0. \end{aligned}$$

Moreover, *A* is monotone on $(0,c^{1/p})$. Assuming to the contrary that $|u_-| \ge u_+$, we would have that $A(|u_-|)\ge A(u_+)$ and hence $A(u_+)\le A(|u_-|) < A(u_-)$, which contradicts the fact that $A(u_+)=A(u_-)$. Hence, $0<|u_-|<u_+<c^{1/p}$, which implies that $|I_1(E)|<I_2(E)$, and therefore, $T'(E)<0$ also in the case when *p* is odd. The proof of Proposition 1 is complete. $\square$

The following result describes the limiting points of the energy-to-period map $E \mapsto 2T(E)$ and is proved with routine computations.

### Lemma 2

For every $c > 0$ and $p \in {\mathbb {N}}$, let $E \mapsto 2T(E)$ be the mapping defined by (12). Then

23 $$\begin{aligned} T(0) := \lim _{E\rightarrow 0} T(E)= \pi c^{1/2}, \end{aligned}$$

and there exists $T_1 \in (0,\pi )$ such that

24 $$\begin{aligned} T(E_c) := \lim _{E \rightarrow E_c} T(E)= T_1 c^{1/2}, \end{aligned}$$

with $E_c$ defined in (11).

### Proof

We can write (12) in the explicit form

25 $$\begin{aligned} T(E) = \int _{u_-}^{u_+} \frac{\sqrt{B(u)} \mathrm{d}u}{\sqrt{E - A(u)}}, \end{aligned}$$

where the turning points $u_{\pm } \gtrless 0$ are given by the roots of $A(u_{\pm }) = E$. To prove the first assertion, we use the scaling transformation

$$\begin{aligned} u = \left( \frac{2E}{c} \right) ^{1/2} x, \end{aligned}$$

to rewrite the integral in (25) as follows:

$$\begin{aligned} T(E) = c^{1/2} \int _{v_-}^{v_+} \frac{(1 - \mu x^p) \mathrm{d}x}{\sqrt{1 - x^2 + 2 \mu x^{p+2}/(p+2)}}, \quad \mu := \frac{2^{p/2} E^{p/2}}{c^{(p+2)/2}}, \end{aligned}$$

where $v_{\pm } \gtrless 0$ are roots of the algebraic equation

$$\begin{aligned} v_{\pm }^2 = 1 + \frac{2}{p+2} \mu v_{\pm }^{p+2}. \end{aligned}$$

We note that $\mu \rightarrow 0,\,v_{\pm } \rightarrow \pm 1$ as $E \rightarrow 0$, which gives the formal limit

$$\begin{aligned} \int _{v_-}^{v_+} \frac{(1 - \mu x^p) \mathrm{d}x}{\sqrt{1 - x^2 + 2 \mu x^{p+2}/(p+2)}} \rightarrow \int _{-1}^1 \frac{\mathrm{d}x}{\sqrt{1-x^2}} = \pi \quad \text{ as } \quad \mu \rightarrow 0. \end{aligned}$$

This yields the limit (23). The justification of the formal limit is performed by rescaling $[v_-,v_+]$ to $[-1,1]$ and by using Lebesgue’s dominated convergence theorem, since the integrand function and its limit as $\mu \rightarrow 0$ are absolutely integrable.

To prove the second assertion, notice that for $E = E_c$, the turning points $u_{\pm }$ used in the integral (25) are known as $u_{\pm } = \pm c^{1/p} q_{\pm }$, where $q_+ = 1$ and $q_- > 0$ is a root of the algebraic equation

$$\begin{aligned} q_-^2 - \frac{2}{p+2} (-1)^p q_-^{p+2} = \frac{p}{p+2}. \end{aligned}$$

If *p* is even, $q_- = 1$, while if *p* is odd, $q_- \in (0,1)$, as follows from the proof of Proposition 1. By splitting the integral (25) into two parts, we integrate over $[u_-,0]$ and $[0,u_+]$ separately and use the substitution $u = \pm c^{1/p} x$ for the two integrals. Since $T'(E)$ is bounded for every $E > 0$ from the representation (20) and is integrable as $E \rightarrow E_c$, we obtain that $T(E_c) := \lim _{E \rightarrow E_c} T(E)$ exists and is given by $T(E_c) = T_1 c^{1/2}$, where

26 $$\begin{aligned} T_1:= & {} \int _{0}^{1} \frac{(1 - x^p) \mathrm{d}x}{\sqrt{1 - x^2 - 2(1 - x^{p+2})/(p+2)}}\nonumber \\&+ \int _{0}^{q_-} \frac{(1 - (-1)^p x^p) \mathrm{d}x}{\sqrt{1 - x^2 - 2(1 - (-1)^p x^{p+2})/(p+2)}}. \end{aligned}$$

Both integrals are finite and positive, from which the existence of $T_1 > 0$ is concluded. Since $T'(E) < 0$ for every $E > 0$, we have that $T_1 < \pi$. $\square$

## Continuation of smooth periodic waves with respect to *c*

**Fig. 2:**
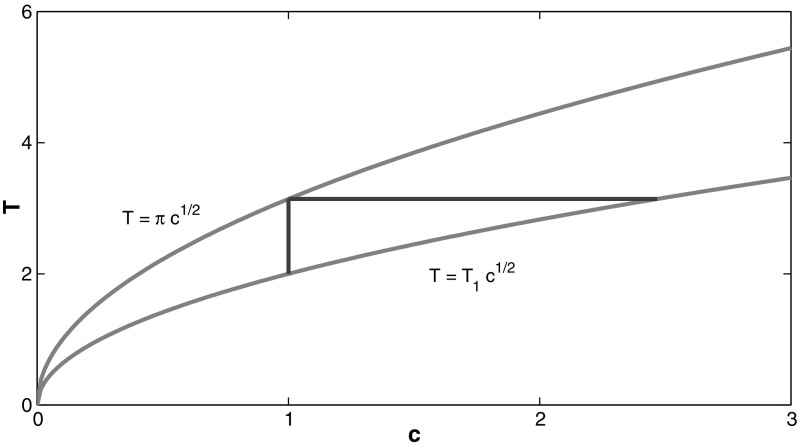
Existence region for smooth periodic waves in the (*T*, *c*) parameter plane between the two limiting curves $T = \pi c^{1/2}$ and $T = T_1 c^{1/2}$ obtained in Lemma 2

In Sect. 2, we fixed the parameter $c > 0$ and considered a continuation of the smooth periodic wave solutions *U* with respect to the parameter *E* in $(0,E_c)$, where $E = 0$ corresponds to the zero solution and $E = E_c$ corresponds to a peaked periodic wave. The mapping $E \mapsto 2T(E)$ is found to be monotonically decreasing according to Proposition 1. Therefore, this mapping can be inverted for every fixed $c > 0$ and we denote the corresponding dependence by *E*(*T*, *c*). The range of the mapping $E \mapsto 2T(E)$, which was calculated in Lemma 2, specifies the domain of the function *E*(*T*, *c*) with respect to the parameter *T* at fixed *c*. The existence interval for the smooth periodic waves between the two limiting cases (23) and (24) obtained in Lemma 2 is shown in Fig. 2.

When we fix the parameter $c > 0$, the half period *T* belongs to the interval $(T_1 c^{1/2},\pi c^{1/2})$, which corresponds to the vertical line in Fig. 2. When we fix the parameter $T > 0$, the parameter *c* belongs to the interval $(T^2/\pi ^2,T^2/T_1^2)$, which corresponds to the horizontal line in Fig. 2.

In this section, we will fix the period 2*T* and consider a continuation of the smooth periodic wave solutions *U* with respect to the parameter *c* in a subset of ${\mathbb {R}}^+$. The next result specifies the interval of existence for the speed *c*.

### Lemma 3

For every $T > 0$ and $p \in {\mathbb {N}}$, there exists a family of 2*T*-periodic solutions $U=U(z;c)$ of Eq. (2) parametrized by $c \in (c_0(T),c_1(T))$, where

27 $$\begin{aligned} c_0(T) := \frac{T^2}{\pi ^2}, \quad c_1(T) := \frac{T^2}{T_1^2} > c_0(T), \end{aligned}$$

with $T_1 \in (0,\pi )$ given in (26) and $U \rightarrow 0$ as $c \rightarrow c_0(T)$. Moreover, the mapping $(c_0(T),c_1(T)) \ni c \mapsto U \in {\dot{L}}_{\mathrm{per}}^2(-T,T) \cap H^{\infty }_{\mathrm{per}}(-T,T)$ is $C^1$.

### Proof

Notice that the scaling transformation

28 $$\begin{aligned} U(z;c) = c^{1/p} {\tilde{U}}({\tilde{z}}), \quad z = c^{1/2} {\tilde{z}}, \quad T = c^{1/2} {\tilde{T}}, \end{aligned}$$

relates 2*T*-periodic solutions *U* of the boundary value problem (2) to $2{\tilde{T}}$-periodic solutions ${\tilde{U}}$ of the same boundary value problem with *c* normalized to 1, that is,

29 $$\begin{aligned} \frac{\mathrm{d}}{\mathrm{d} {{\tilde{z}}}} \left[ (1 - {{\tilde{U}}}^p) \frac{\mathrm{d} {{\tilde{U}}}}{\mathrm{d}{{\tilde{z}}}} \right] + {{\tilde{U}}}({{\tilde{z}}}) = 0, \quad {{\tilde{U}}}(-{{\tilde{T}}}) = {{\tilde{U}}}({{\tilde{T}}}), \quad {{\tilde{U}}}'(-{{\tilde{T}}}) ={{\tilde{U}}}'({{\tilde{T}}}). \end{aligned}$$

Lemma 1 guarantees the existence of a family $\{{{\tilde{U}}}_{\tilde{E}}\}_{\tilde{E} \in (0,E_1)}$ of $2{{\tilde{T}}}({{\tilde{E}}})$-periodic solutions of the boundary value problem (29). In view of Lemma 2 and since *T* is fixed, we have ${{\tilde{T}}}({{\tilde{E}}}) = c^{-1/2}T \in (T_1,\pi )$, which implies that *c* belongs to the interval $(c_0(T),c_1(T))$, where $c_0(T)$ and $c_1(T)$ are given by (27). Moreover, this relation provides a one-to-one correspondence between the parameters *c* and ${{\tilde{E}}}$ in view of the fact that ${{\tilde{T}}}'({{\tilde{E}}})<0$ by Proposition 1 which implies that $c^{1/2} =T/{{\tilde{T}}} ({{\tilde{E}}})$ is monotone increasing in ${{\tilde{E}}}$. In view of the transformation (28), we therefore obtain existence of a family $\{U_c\}_{c \in (c_0(T),c_1(T))}$ of 2*T*-periodic solutions of the boundary value problem (2). The value $c_0(T)$ corresponds to the zero solution, whereas $c_1(T)$ corresponds to the peaked periodic wave. $\square$

Recall that the mapping $E \mapsto 2T(E)$ can be inverted for every fixed $c > 0$ and that the corresponding dependence is denoted by *E*(*T*, *c*). The next result shows that *E*(*T*, *c*) is a monotonically increasing function of $c\in (c_0(T),c_1(T))$ for every fixed $T > 0$.

### Lemma 4

For every $T > 0,\,p \in {\mathbb {N}}$, the mapping $(c_0(T),c_1(T)) \ni c \mapsto E(T,c)$ is $C^1$ and monotonically increasing.

### Proof

Using the transformation (28) in the boundary value problem (29), we obtain that

$$\begin{aligned} E(T,c) = c^{\frac{p+2}{p}} {\tilde{E}}, \end{aligned}$$

where ${\tilde{E}}$ is the energy level of the first integral of the second-order equation in (29),

$$\begin{aligned} {\tilde{E}} = \frac{1}{2} (1 - {{\tilde{U}}}^p)^2 \left( \frac{\mathrm{d}{\tilde{U}}}{\mathrm{d}{\tilde{z}}} \right) ^2 + \frac{1}{2} {{\tilde{U}}}^2 - \frac{1}{p+2} {{\tilde{U}}}^{p+2}. \end{aligned}$$

Now, as *T* is fixed and ${\tilde{T}} = {\tilde{T}}({\tilde{E}})$ is defined by (12) for *c* normalized to 1, we can define *E*(*T*, *c*) from the root of the following equation

30 $$\begin{aligned} T = c^{\frac{1}{2}} {\tilde{T}}\left( E(T,c) c^{-\frac{p+2}{p}}\right) . \end{aligned}$$

Since ${\tilde{T}}(0) = \pi$ and ${\tilde{T}}(E_1) = T_1$, we have roots $E(T,c_0(T)) = 0$ and $E(T,c_1(T)) = E_c$ of the algebraic equation (30), with $E_c$ given by (11) at $c = c_1(T)$. In order to continue the roots by using the implicit function theorem for every $c \in (c_0(T),c_1(T))$, we differentiate (30) with respect to *c* at fixed *T* and obtain

31 $$\begin{aligned} 0 = \frac{1}{2} c^{-\frac{1}{2}} {\tilde{T}}({\tilde{E}}) - \frac{p+2}{p} E c^{-\frac{3p+4}{2p}} {\tilde{T}}'({\tilde{E}}) + c^{-\frac{p+4}{2 p}} {\tilde{T}}'({\tilde{E}}) \frac{\partial E(T,c)}{\partial c}. \end{aligned}$$

By Proposition 1, we have ${\tilde{T}}'({\tilde{E}}) < 0$ for ${\tilde{E}} \in (0,E_1)$, so that we can rewrite (31) as follows:

32 $$\begin{aligned} \left| {\tilde{T}}'({\tilde{E}}) \right| \frac{\partial E(T,c)}{\partial c} = \frac{1}{2} c^{\frac{2}{p}} {\tilde{T}}({\tilde{E}}) + \frac{p+2}{p} E c^{-1} \left| {\tilde{T}}'({\tilde{E}}) \right| > 0. \end{aligned}$$

Recall that ${\tilde{T}}'({\tilde{E}})$ is nonzero for every $\tilde{E} \in (0,E_1)$ and in the limit $\tilde{E} \rightarrow E_1$. By the implicit function theorem and thanks to the smoothness of all dependencies, there exists a unique, monotonically increasing $C^1$ map $(c_0(T),c_1(T)) \ni c \mapsto E(T,c)$ such that *E*(*T*, *c*) is a root of Eq. (30) and $E(T,c_1(T)) = E_c$, where $E_c$ is given by (11) at $c = c_1(T)$. $\square$

We shall now consider how the $L^2_{\mathrm{per}}(-T,T)$ norm of the periodic wave *U* with fixed period 2*T* depends on the parameter *c*. In order to prove that it is an increasing function of *c* in $(c_0(T),c_1(T))$, we obtain a number of identities satisfied by the periodic wave *U*. This result will be used in the proof of Proposition 3 in Sect. 5.

### Lemma 5

For every $T > 0,\,p \in {\mathbb {N}}$, the mapping $(c_0(T),c_1(T)) \ni c \mapsto \Vert U \Vert _{L^2_{\mathrm{per}}(-T,T)}^2$ is $C^1$ and monotonically increasing. Moreover, if the operator *L* is defined by (4), then $\partial _c U \in {\dot{L}}_{\mathrm{per}}^2(-T,T)$ satisfies

33 $$\begin{aligned} L \partial _c U = -U \end{aligned}$$

and

34 $$\begin{aligned} \langle \partial _c U,U\rangle _{L^2_{\mathrm{per}}} > 0. \end{aligned}$$

### Proof

Integrating (2) in *z* with zero mean, we can write

35 $$\begin{aligned} (c-U^p) \partial _z U + \partial _z^{-1} U = 0. \end{aligned}$$

From here, multiplication by $\partial _z^{-1} U$ and integration by parts yield

36 $$\begin{aligned} \Vert \partial _z^{-1} U \Vert ^2_{L^2_{\mathrm{per}}(-T,T)} = c \Vert U \Vert _{L^2_{\mathrm{per}}(-T,T)}^2 - \frac{1}{p+1} \int _{-T}^T U^{p+2} \mathrm{d}z. \end{aligned}$$

On the other hand, integrating (3) over the period 2*T* and using Eqs. (35) and (36) yield

37 $$\begin{aligned} 2 E(T,c) T= & {} \frac{c}{2} \Vert U \Vert _{L^2_{\mathrm{per}}(-T,T)}^2 - \frac{1}{p+2} \int _{-T}^T U^{p+2} \mathrm{d}z + \frac{1}{2} \left\| (c-U^p) \frac{\mathrm{d}U}{\mathrm{d}z} \right\| ^2_{L^2_{\mathrm{per}}(-T,T)} \nonumber \\= & {} \frac{c}{2} \Vert U \Vert _{L^2_{\mathrm{per}}(-T,T)}^2 - \frac{1}{p+2} \int _{-T}^T U^{p+2} \mathrm{d}z + \frac{1}{2} \Vert \partial _z^{-1} U \Vert ^2_{L^2_{\mathrm{per}}(-T,T)}\nonumber \\= & {} c \Vert U \Vert _{L^2_{\mathrm{per}}(-T,T)}^2 - \frac{(3p+4)}{2(p+1)(p+2)} \int _{-T}^T U^{p+2} \mathrm{d}z. \end{aligned}$$

Expressing $c \Vert U \Vert _{L^2_{\mathrm{per}}(-T,T)}^2$ from Eqs. (36) and (37), we obtain

38 $$\begin{aligned} \Vert \partial _z^{-1} U \Vert ^2_{L^2_{\mathrm{per}}} = 2 E(T,c) T + \frac{p}{2(p+1)(p+2)} \int _{-T}^T U^{p+2} dz. \end{aligned}$$

From the fact that *U* is a critical point of $H(u) + c Q(u)$ given by (5) and (7) for a fixed period 2*T*, we obtain

39 $$\begin{aligned} \frac{\mathrm{d}{\mathcal {H}}}{\mathrm{d}c} + c \frac{\mathrm{d}{\mathcal {Q}}}{\mathrm{d}c} = 0, \end{aligned}$$

where

40 $$\begin{aligned} {\mathcal {H}}(c)= & {} - \frac{1}{2} \Vert \partial _z^{-1} U \Vert ^2_{L^2_{\mathrm{per}}(-T,T)} - \frac{1}{(p+1)(p+2)} \int _{-T}^T U^{p+2} \mathrm{d}z \nonumber \\= & {} -E(T,c) T - \frac{(p+4)}{4(p+1)(p+2)}\int _{-T}^T U^{p+2} \mathrm{d}z \end{aligned}$$

and

41 $$\begin{aligned} c {\mathcal {Q}}(c)= & {} \frac{c}{2} \Vert U \Vert ^2_{L^2_{\mathrm{per}}(-T,T)} \nonumber \\= & {} E(T,c) T + \frac{(3p+4)}{4(p+1)(p+2)} \int _{-T}^T U^{p+2} \mathrm{d}z \end{aligned}$$

are simplified with the help of Eqs. (37) and (38) again. Next, we differentiate (40) and (41) in *c* for fixed *T* and use (39) to obtain the constraint

42 $$\begin{aligned} \frac{\mathrm{d}{\mathcal {H}}}{\mathrm{d}c} + c \frac{\mathrm{d}{\mathcal {Q}}}{\mathrm{d}c}= & {} - \frac{(p+4)}{4(p+1)(p+2)} \frac{\mathrm{d}}{\mathrm{d}c} \int _{-T}^T U^{p+2} \mathrm{d}z - {\mathcal {Q}}(c)\nonumber \\&+\,\frac{(3p+4)}{4(p+1)(p+2)} \frac{\mathrm{d}}{\mathrm{d}c} \int _{-T}^T U^{p+2} \mathrm{d}z \nonumber \\= & {} - {\mathcal {Q}}(c) + \frac{p}{2(p+1)(p+2)} \frac{\mathrm{d}}{\mathrm{d}c} \int _{-T}^T U^{p+2} \mathrm{d}z = 0. \end{aligned}$$

From (32), (39), (40) and (42), we finally obtain

43 $$\begin{aligned} c \frac{\mathrm{d}{\mathcal {Q}}}{\mathrm{d}c} = -\frac{\mathrm{d} {\mathcal {H}}}{\mathrm{d}c}= & {} T \frac{\partial E(T,c)}{\partial c} + \frac{(p+4)}{4(p+1)(p+2)} \frac{\mathrm{d}}{\mathrm{d}c} \int _{-T}^T U^{p+2} \mathrm{d}z \nonumber \\= & {} T \frac{\partial E(T,c)}{\partial c} + \frac{p+4}{2p} {\mathcal {Q}}(c) > 0. \end{aligned}$$

To prove the second assertion, recall that the family of periodic waves *U*(*z*; *c*) is $C^1$ with respect to *c* by Lemma 3. Differentiating the second-order equation in (2) with respect to *c* at fixed period 2*T* and integrating it twice with zero mean yields Eq. (33). Notice that $\partial _c U$ is again 2*T*-periodic, since the period of *U* is fixed independently of *c*. Finally, we find that

$$\begin{aligned} \langle \partial _c U, U \rangle _{L^2_{\mathrm{per}}} = \frac{1}{2} \frac{\mathrm{d}}{\mathrm{d}c} \Vert U \Vert _{L^2_{\mathrm{per}}}^2 >0, \end{aligned}$$

since by the first assertion, the mapping $c\mapsto \Vert U \Vert _{L^2_{\mathrm{per}}}^2$ is monotonically increasing. $\square$

As an immediate consequence of Lemmas 3 and 5, we prove the following result which will be used in the proof of Proposition 2 in Sect. 4.

### Corollary 1

For every $T > 0,\,p \in {\mathbb {N}}$ and $c\in (c_0(T),c_1(T))$, the periodic solution *U* of the boundary value problem (2) satisfies

44 $$\begin{aligned} \int _{-T}^T U^{p+2} \mathrm{d}z > 0. \end{aligned}$$

### Proof

It follows from (42) that

45 $$\begin{aligned} \frac{\mathrm{d}}{\mathrm{d}c} \int _{-T}^T U^{p+2} \mathrm{d}z = \frac{2(p+1)(p+2)}{p} {\mathcal {Q}}(c) > 0, \quad c \in (c_0(T),c_1(T)). \end{aligned}$$

On the other hand, $\int _{-T}^T U^{p+2} \mathrm{d}z = 0$ at $c = c_0(T)$ by Lemma 3. Integrating the inequality (45) for $c > c_0(T)$ implies positivity of $\int _{-T}^T U^{p+2} \mathrm{d}z$. $\square$

## Negative index of the operator *L*

Recall that $T(E) \rightarrow T(0) = \pi c^{1/2}$ and $U\rightarrow 0$ as $E \rightarrow 0$ in view of Lemma 2. In this limit, the operator given by (4) becomes an integral operator with constant coefficients,

$$\begin{aligned} L_0 = P_0( \partial _z^{-2} + c)P_0{:}\,\dot{L}_{\mathrm{per}}^2(-T(0),T(0)) \rightarrow \dot{L}_{\mathrm{per}}^2(-T(0),T(0)), \end{aligned}$$

whose spectrum can be computed explicitly as

46 $$\begin{aligned} \sigma (L_0) = \left\{ c(1 - n^{-2}), \quad n \in {\mathbb {Z}} {\backslash } \{0\} \right\} , \end{aligned}$$

by using Fourier series. For every $c > 0$, the spectrum of $L_0$ is purely discrete and consists of double eigenvalues accumulating to the point *c*. All double eigenvalues are strictly positive except for the lowest eigenvalue, which is located at the origin. As is shown in [15] with a perturbation argument for $p = 1$ and $p = 2$, the spectrum of *L* for *E* near 0 includes a simple negative eigenvalue, a simple zero eigenvalue, and the positive spectrum is bounded away from zero. We will show that this conclusion remains true for the entire family of smooth periodic waves. Let us first prove the following.

### Lemma 6

For every $c > 0,\,p \in {\mathbb {N}}$, and $E \in (0,E_c)$, the operator *L* given by (4) is self-adjoint and its spectrum includes a countable set of isolated eigenvalues below

47 $$\begin{aligned} C_-(E) := \inf _{z \in [-T(E),T(E)]} (c - U(z)^p) > 0. \end{aligned}$$

### Proof

The self-adjoint properties of *L* are obvious. For every $E \in (0,E_c)$, there are positive constants $C_{\pm }(E)$ such that

48 $$\begin{aligned} C_-(E) \le c - U(z)^p \le C_+(E) \quad \text{ for } \text{ every } \;\; z \in [-T(E),T(E)]. \end{aligned}$$

For the rest of the proof we use the short notation $T = T(E)$. The eigenvalue equation $(L-\lambda I) v = 0$ for $v \in {\dot{L}}^2_{\mathrm{per}}(-T,T)$ is equivalent to the spectral problem

49 $$\begin{aligned} P_0 (c-U^p-\lambda ) P_0 v = - P_0 \partial _z^{-2} P_0 v. \end{aligned}$$

Under the condition $\lambda < C_-(E)$, we have $c - U^p - \lambda \ge C_-(E) - \lambda > 0$. Setting

50 $$\begin{aligned} w := (c-U^p-\lambda )^{1/2} P_0 v \in L^2_{\mathrm{per}}(-T,T), \quad \lambda < C_-(E), \end{aligned}$$

we find that $\lambda$ is an eigenvalue of the spectral problem (49) if and only if 1 is an eigenvalue of the self-adjoint operator

51 $$\begin{aligned}&K(\lambda ) = - (c-U^p-\lambda )^{-1/2} P_0 \partial _z^{-2} P_0 (c-U^p-\lambda )^{-1/2}:\nonumber \\&\quad \qquad \qquad L^2_{\mathrm{per}}(-T,T) \rightarrow L^2_{\mathrm{per}}(-T,T), \end{aligned}$$

that is,^5^ $w = K(\lambda ) w$. The operator $K(\lambda )$ for every $\lambda < C_-(E)$ is a compact (Hilbert–Schmidt) operator thanks to the bounds (48) and the compactness of $P_0 \partial _z^{-2} P_0$. Consequently, the spectrum of $K(\lambda )$ in $L^2_{\mathrm{per}}(-T,T)$ for every $\lambda < C_-(E)$ is purely discrete and consists of isolated eigenvalues. Moreover, these eigenvalues are positive thanks to the positivity of $K(\lambda )$, as follows:

52 $$\begin{aligned} \langle K(\lambda ) w, w \rangle _{L^2_{\mathrm{per}}} = \Vert P_0 \partial _z^{-1} P_0 (c-U^p-\lambda )^{-1/2} w \Vert _{L^2_{\mathrm{per}}}^2 \ge 0, \quad \forall w \in L^2_{\mathrm{per}}(-T,T). \end{aligned}$$

We note that

- $$K(\lambda ) \rightarrow 0^+$$ as $$\lambda \rightarrow -\infty $$,
- $$K'(\lambda ) > 0$$ for every $$\lambda < C_-(E)$$.

Claim (a) follows from (52) via spectral calculus:

$$\begin{aligned} \langle K(\lambda ) w, w \rangle _{L^2_{\mathrm{per}}} \sim |\lambda |^{-1} \Vert P_0 \partial _z^{-1} P_0 w \Vert _{L^2}^2 \quad \text{ as } \quad \lambda \rightarrow -\infty . \end{aligned}$$

Claim (b) follows from the differentiation of $K(\lambda )$,

$$\begin{aligned} \langle K'(\lambda ) w, w \rangle _{L^2_{\mathrm{per}}}= & {} \frac{1}{2} \langle \rho (\lambda )K(\lambda )w,w\rangle _{L^2_{\mathrm{per}}} +\frac{1}{2} \left\langle K(\lambda ) \rho (\lambda )w,w\right\rangle _{L^2_{\mathrm{per}}}, \end{aligned}$$

where we have defined the weight function $\rho (\lambda ) := (c - U^p - \lambda )^{-1}$ which is strictly positive and uniformly bounded thanks to (48). Since $K(\lambda )$ is positive due to (52), both terms in the above expression are positive in view of a generalization of Sylvester’s law of inertia for differential operators; see Theorem 4.2 in [23]. Indeed, to prove that the first term is positive it suffices to show that the eigenvalues $\mu$ of $\rho (\lambda ) K(\lambda )$ are positive. The corresponding spectral problem $\rho (\lambda ) K(\lambda ) w = \mu w$ is equivalent to $\rho (\lambda )^{1/2} K(\lambda ) \rho (\lambda )^{1/2} v = \mu v$ in view of the substitution $w=\rho (\lambda )^{1/2}v$. By Sylvester’s law, the number of negative eigenvalues of $K(\lambda )$ is equal to the number of negative eigenvalues of the congruent operator ${{\tilde{K}}}(\lambda ) = \rho (\lambda )^{1/2} K(\lambda ) \rho (\lambda )^{1/2}$. Therefore, $\rho (\lambda )K(\lambda )$ is positive in view of the positivity of $K(\lambda )$. The second term can be treated in the same way.

It follows from claims (a) and (b) that positive isolated eigenvalues of $K(\lambda )$ are monotonically increasing functions of $\lambda$ from the zero level as $\lambda \rightarrow -\infty$. The location and number of crossings of these eigenvalues with the unit level give the location and number of eigenvalues $\lambda$ in the spectral problem (49). The compactness of $K(\lambda )$ for $\lambda < C_-(E)$ therefore implies that there exists a countable (finite or infinite) set of isolated eigenvalues of *L* below $C_-(E)$. $\square$

Next, we inspect analytical properties of eigenvectors for isolated eigenvalues below $C_-(E) > 0$ given by (47).

### Lemma 7

Under the condition of Lemma 6, let $\lambda _0 < C_-(E)$ be an eigenvalue of the operator *L* given by (4). Then, $\lambda _0$ is at most double and the eigenvector $v_0$ belongs to ${\dot{L}}^2_{\mathrm{per}}(-T(E),T(E)) \cap H^{\infty }_{\mathrm{per}}(-T(E),T(E))$.

### Proof

As in the proof of the previous Lemma, we use the shorthand $T = T(E)$ for lighter notation. The eigenvector $v_0 \in {\dot{L}}^2_{\mathrm{per}}(-T,T)$ for the eigenvalue $\lambda _0 < C_-(E)$ satisfies the spectral problem (49) written as the integral equation

53 $$\begin{aligned} P_0 \partial _z^{-2}P_0 v_0 + P_0 (c-U^p-\lambda _0)P_0 v_0 = 0. \end{aligned}$$

Since $U \in H^{\infty }_{\mathrm{per}}(-T,T)$ and $c - U^p - \lambda _0 \ge C_-(E) - \lambda _0 > 0$, we obtain that $v_0\in H^{2}_{\mathrm{per}}(-T,T)$, and by bootstrapping arguments we find that $v_0 \in H^{\infty }_{\mathrm{per}}(-T,T)$. Applying two derivatives to the integral equation (53), we obtain the equivalent differential equation for the eigenvector $v_0 \in {\dot{L}}^2_{\mathrm{per}}(-T,T) \cap H^{\infty }_{\mathrm{per}}(-T,T)$ and the eigenvalue $\lambda _0 < C_-(E)$:

54 $$\begin{aligned} v_0 +\partial _z^2 \left[ (c-U^p - \lambda _0) v_0 \right] = 0. \end{aligned}$$

The second-order differential equation (54) admits at most two linearly independent solutions in ${\dot{L}}^2_{\mathrm{per}}(-T,T)$ and so does the integral equation (53) for an eigenvalue $\lambda _0 < C_-(E)$. Since *L* is self-adjoint, the eigenvalue $\lambda _0$ is not defective,^6^ and hence, the multiplicity of $\lambda _0$ is at most two. $\square$

We are now ready to prove the main result of this section. This proves part (b) of Theorem 1.

### Proposition 2

For every $c > 0,\,p \in {\mathbb {N}}$, and $E \in (0,E_c)$, the operator *L* given by (4) has exactly one simple negative eigenvalue, a simple zero eigenvalue, and the rest of the spectrum is positive and bounded away from zero.

### Proof

Thanks to Lemma 6, we only need to inspect the multiplicity of negative and zero eigenvalues of *L*. By Lemma 7, the zero eigenvalue $\lambda _0 = 0 < C_-(E)$ can be at most double. The first eigenvector $v_0 = \partial _z U \in {\dot{L}}^2_{\mathrm{per}}(-T(E),T(E)) \cap H^{\infty }_{\mathrm{per}}(-T(E),T(E))$ for $\lambda _0 = 0$ follows by the translational symmetry. Indeed, differentiating (2) with respect to *z*, we verify that $v_0$ satisfies the differential equation (54) with $\lambda _0 = 0$ and, equivalently, the integral equation (53) with $\lambda _0 = 0$.

Another linearly independent solution $v_1 = \partial _E U$ of the same Eq. (54) with $\lambda _0 = 0$ is obtained by differentiating (2) with respect to *E* for fixed $c>0$. Here we understand the family *U*(*z*; *E*) of smooth 2*T*(*E*)-periodic solutions constructed in Lemma 1, where the period 2*T*(*E*) is given by (12) and is a smooth function of *E*. Now, we show that the second solution $v_1$ is not 2*T*(*E*)-periodic under the condition $T'(E) < 0$ established in Proposition 1. Consequently, the zero eigenvalue $\lambda _0 = 0$ is simple. For simplicity, we assume that the family *U*(*z*; *E*) satisfies the condition

55 $$\begin{aligned} U(\pm T(E);E) = 0 \end{aligned}$$

at the end points, which can be fixed by translational symmetry. By differentiating the first boundary condition in (2) with respect to *E*, we obtain

$$\begin{aligned} \partial _E U(-T(E);E) - T'(E) \partial _z U(-T(E);E) = \partial _E U(T(E);E) + T'(E) \partial _z U(T(E);E). \end{aligned}$$

Notice that $\partial _z U(\pm T(E);E) \ne 0$, since otherwise the periodic solution *U* would be identically zero in view of (55) which is only possible for $E = 0$. Since $T'(E) \ne 0$ by Proposition 1, the solution $v_1 = \partial _E U$ is not 2*T*(*E*)-periodic, and therefore, the zero eigenvalue $\lambda _0 = 0$ is simple for the entire family of smooth *T*(*E*)-periodic solutions.

Next, we show that the spectrum of *L* includes at least one negative eigenvalue. Indeed, from the integral version of the differential equation (2),

$$\begin{aligned} P_0 \left( c - \frac{1}{p+1} U^p \right) P_0 U + P_0 \partial _z^{-2} P_0 U = 0, \end{aligned}$$

we obtain that $LU = -\frac{p}{p+1} P_0 U^{p+1}$, which implies that

56 $$\begin{aligned} \langle LU , U \rangle _{L^2_{\mathrm{per}}} = -\frac{p}{p+1} \int _{-T(E)}^{T(E)} U^{p+2} \mathrm{d}z < 0. \end{aligned}$$

The last inequality is obvious for even *p*. For odd *p* it follows from Corollary 1 for given $T(E) \in (T_1 c^{1/2},\pi c^{1/2})$ fixed. In both cases, we have shown that *L* has at least one negative eigenvalue for every $E \in (0,E_c)$.

Finally, the spectrum of *L* includes at most one simple negative eigenvalue. Indeed, the family of 2*T*(*E*)-periodic solutions is smooth with respect to the parameter $E \in (0,E_c)$ and it reduces to the zero solution as $E\rightarrow 0$. It follows from the spectrum (46) for the operator $L_0$ at the zero solution, and the preservation of the simple zero eigenvalue with the eigenvector $\partial _z U$ for every $E \in (0,E_c)$, that the splitting of a double zero eigenvalue for $E \ne 0$ results in appearance of at most one negative eigenvalue of *L*. Thus, there exists exactly one simple negative eigenvalue of *L* for every $E \in (0,E_c)$. $\square$

## Applications of the Hamilton–Krein theorem

Since *L* has a simple zero eigenvalue in ${\dot{L}}^2_{\mathrm{per}}(-T,T)$ by Proposition 2 with the eigenvector $v_0 = \partial _z U$, eigenvectors $v \in {\dot{H}}^1_{\mathrm{per}}(-T,T)$ of the spectral problem $\lambda v = \partial _z Lv$ for nonzero eigenvalues $\lambda$ satisfy the constraint $\langle U, v \rangle _{L^2_{\mathrm{per}}} = 0$; see definition (8) of the space $L^2_c$. Since $\partial _z$ is invertible in space ${\dot{L}}^2_{\mathrm{per}}(-T,T)$ and the inverse operator is bounded from ${\dot{L}}^2_{\mathrm{per}}(-T,T)$ to itself, we can rewrite the spectral problem $\lambda v = \partial _z Lv$ in the equivalent form

57 $$\begin{aligned} \lambda P_0 \partial _z^{-1} P_0 v = L v, \quad v \in {\dot{L}}^2_{\mathrm{per}}(-T,T). \end{aligned}$$

In this form, the Hamilton–Krein theorem from [12] applies directly in $L^2_c$. According to this theorem, the number of unstable eigenvalues with $\lambda \notin i {\mathbb {R}}$ is bounded by the number of negative eigenvalues of *L* in the constrained space $L^2_c$. Therefore, we only need to show that the operator *L* is positive in $L^2_c$ with only a simple zero eigenvalue due to the translational invariance in order to prove part (c) of Theorem 1. The corresponding result is given by the following proposition.

### Proposition 3

For every $c > 0,\,p \in {\mathbb {N}}$, and $E \in (0,E_c)$, the operator $L |_{L^2_c}{:}\,L^2_c \rightarrow L^2_c$, where *L* is given by (4), has a simple zero eigenvalue and a positive spectrum bounded away from zero.

### Proof

The proof relies on a well-known criterion (see for example Lemma 1 in [11] or Theorem 4.1 in [23]) which ensures positivity of the self-adjoint operator *L* with properties obtained in Proposition 2, when it is restricted to a co-dimension one subspace. Positivity of $L |_{L^2_c}{:}\,L^2_c \rightarrow L^2_c$ is achieved under the condition

58 $$\begin{aligned} \langle L^{-1} U,U\rangle _{L^2_{\mathrm{per}}} <0. \end{aligned}$$

To show (58), we observe that $\mathrm{Ker}(L) = \mathrm{span}\{ v_0\}$, where $v_0=\partial _z U$ and $\langle U, v_0 \rangle _{L^2_{\mathrm{per}}} = 0$ implies that $U \in \mathrm{Ker}(L)^{\perp }$. By Fredholm’s alternative (see, for example, Theorem B.4 in [23]), $L^{-1} U$ exists in ${\dot{L}}_{\mathrm{per}}^2(-T,T)$ and can be made unique by the orthogonality condition $\langle L^{-1} U, v_0 \rangle _{L^2_{\mathrm{per}}} = 0$. By Lemma 5, we have the existence of $\partial _c U \in {\dot{L}}_{\mathrm{per}}^2(-T,T)$ such that $L \partial _c U =-U$, see Eq. (33). Moreover, $\langle \partial _c U, v_0 \rangle _{L^2_{\mathrm{per}}} =0$, since $\partial _c U$ and $v_0=\partial _z U$ have opposite parity. Therefore, $\partial _c U = L^{-1} U$ and we obtain

$$\begin{aligned} \langle L^{-1} U,U\rangle _{L^2_{\mathrm{per}}} = - \langle \partial _c U,U \rangle _{L^2_{\mathrm{per}}} <0, \end{aligned}$$

where the strict negativity follows from Lemma 5. $\square$

The proof of Theorem 1 follows from the results of Propositions 1, 2 and 3.

## Footnotes

1 Note that this transformation reflects the change of coordinates owing to which the reduced Ostrovsky equations are reduced to the semi-linear equations of the Klein–Gordon type. This transformation also changes the period of the travelling periodic wave.

2 The largest punctured neighbourhood of a centre which consists entirely of periodic orbits is called *period annulus*; see [2].

3 The idea for this approach of resolving the singularity is taken from [5, Lemma 4.1], where the authors prove a more general result for polynomial systems having first integrals of the form (10).

4 Note that (18) also follows by applying Gelfand–Leray derivatives in (17); see [13, Theorem 26.32, p. 526].

5 This reformulation can be viewed as an adjoint version of the Birmann–Schwinger principle used in analysis of isolated eigenvalues of Schrödinger operators with rapidly decaying potentials [9].

6 Recall that the eigenvalue is called defective if its algebraic multiplicity exceeds its geometric multiplicity.
